# Supplementary material for: Implications of nasal delivery of bromelain on its pharmacokinetics, tissue distribution and pharmacodynamic profile—A preclinical study
Source: PLoS One. 2022 Nov 18;17(11):e0277849. doi: 10.1371/journal.pone.0277849 (PMC9674142; doi:10.1371/journal.pone.0277849)
Supplement: Attachment — Submitted filename: Response to reviewers.doc [file pone.0277849.s001.doc]

**Academic Editor Comments**

**Comment 1:** Please ensure that your manuscript meets PLOS ONE's style requirements, including those for file naming. The PLOS ONE style templates can be found at 
<https://journals.plos.org/plosone/s/file?id=wjVg/PLOSOne_formatting_sample_main_body.pdf> and 
<https://journals.plos.org/plosone/s/file?id=ba62/PLOSOne_formatting_sample_title_authors_affiliations.pdf>

**Response:** We ensure that our revised manuscript meets PLOS ONE’s style requirements, including those for file naming.

**Comment 2:** To comply with PLOS ONE submissions requirements, in your Methods section, please provide additional information regarding the experiments involving animals and ensure you have included details on (1) methods of sacrifice, (2) methods of anesthesia and/or analgesia, and (3) efforts to alleviate suffering.

**Response:** Additional information regarding the experiments involving animals have been included in Method section in detail in revised manuscript as per suggestion made by Academic Editor.

**Comment 3:** Please make modifications to this manuscript to indicate that it is a preliminary study. The number of animal subjects overall and animal subjects per time point are small which doesn't really allow for assessment of differences. Please address this with your revision.

**Response:** As per editor’s suggestion we have made changes in manuscript title and content to indicate that it is a preliminary study. We have performed further studies to derive data on sufficiently large number of animal subjects overall and animal subjects per time point for assessment of differences. The results have been included in revised manuscript.
**Response to reviewers’ comments**

**Reviewer #1:** The PK and tissue distribution of bromelain were conducted and compared in rats following oral and nasal administration, and the pharmacodynamic profile was determined in guinea pigs. The present study is within the scope of PLOS ONE. HOWEVER, the writing elements need further improvement to enhance the quality of the manuscript, and copy editing is beyond the scope of a reviewer’s charge. The authors should pay more attention to further polishing this manuscript. Some of my specific comments are listed below:

**Comment 1:** The animals used in this study (rats and guinea pigs) should be added to both the title and abstract. Otherwise, readers will be misled into thinking that this study was conducted on human patients.

**Response:** In order to prevent the wrong conception about the study by readers, correction in the title and abstract has been made as per reviewer’s suggestion. Title has been changed to “Implications of nasal delivery of bromelain on its pharmacokinetics, tissue distribution and pharmacodynamic profile- a preclinical study.”

Similarly, changes has been made in abstract in revised manuscript i.e., “The present study investigated the preclinical prospects of nasal administration of bromelain on systemic bioavailability, tissue distribution and it’s in vivo anti-histaminic, bronchodilator and anti-asthmatic activity in animal models.”

**Comment 2:** 2. 2.3. Pharmacokinetic studies: To this reviewer’s knowledge, six rats were not enough in a PK study. Additionally, where were the blood samples collected? How many milliliters of blood were collected each time? All these details should be given in the revision.

**Response:** As per reviewer’s suggestion, twelve Wistar rats per group were taken in PK study. Blood samples (300 µl) were collected from rat tail vein in heparinised coated tubes at pre-dose (0.0), 0.5, 1, 2, 4 and 6 h post dose of bromelain solution.

These details have been in incorporated in revised manuscript as “Twenty four Wistar rats were randomly distributed into two groups with twelve animals in each group. Animals of group I received bromelain solution orally (400 µl, 40 mg/Kg body weight) by oral gavage while second group received 30 µl, bromelain solution (10 mg/ Kg body weight) in each nostril in 30 s with the help of micropipette with 0.1 mm internal diameter at delivery place. Blood samples (300 µl) were collected from rat tail vein in heparinised coated tubes at pre-dose (0.0), 0.5, 1, 2, 4 and 6 h post dose of bromelain solution.

**Comment 3:** Three animals per group were sacrificed at 0.5, 1, 2, 4 and 6 h post dosing. Likewise, three rats per time point were not sufficient. These data may not be sufficient to describe inter-individual differences.

**Response:** As per reviewer’s suggestion, number of animals was increased to six at each time point i.e., six animals per group were sacrificed at 0.5, 1, 2, 4 and 6 h post dosing. The results of tissue distribution study at each time point is average of six readings obtained from six animals sacrificed at respective time.

The corrections have been incorporated in revised manuscript under Tissue distribution study as “Sixty healthy wistar rats were randomized into two groups (n= 30). Rats of group I received bromelain solution orally (40 mg/kg body weight) while group II received bromelain solution nasally (10 mg/kg body weight) in each nostril with the help of micropipette with 0.1 mm internal diameter respectively. Six animals per group were sacrificed at 0.5, 1, 2, 4 and 6 h post dosing.

**Comment 4:** Animals were exposed to an aerosol of … The authors should pay more attention to English.

**Response:** As per reviewer’s suggestion, correction has been incorporated in revised manuscript i.e., “Animals were challenged with histamine dihydrochloride aerosol (0.5% w/v) using a nebulizer at a pressure of 300 mm Hg in an air tight Plexiglass chamber (24 × 14 × 24 cm) for 1 min. Consequently after histamine exposure, animals were closely observed to record the pre-convulsive dyspnea time and convulsion time for 9 minutes.”

**Comment 5:** Table 1: Which parameters were statistically significantly different (P<0.05)?

**Response:** All the pharmacokinetic parameters like Cmax, Tmax, k_e_, t_1/2_, MRT and AUC_0-24h_ were statistically significantly different (p<0.05). The same has been highlighted in Table 1.
**Comment 6:** Figure 1B: This reviewer suggests the authors change the style of Figure 1B to Figure 1A.

**Response:** As per reviewer’s suggestion, style of Figure 1B has been changed to Figure 1A.

**Reviewer #2:**

**Comment 1:** Abstract – The meaning of “unforeseeable prophylactic hypersensitivity due to its denaturation or agglomeration hinders its oral use” is unclear.

**Response:** In order to have more clarity changes has been made in abstract as per reviewer’s suggestion.

The changes made are presented in revised manuscript as “However, poor stability in gastric milieu, high dose and immunogenicity associated with prolonged use hinders its oral use.”

**Comment 2:** Section 2.4 – For the oral dose of bromelain solution (40 mg/kg), what is the volume of administration? Is the drug in a solution or in a suspension? What is the vehicle of the solution? Is there any pH adjustment?

The same questions can be applied to the nasal dose solution (10 mg/kg).
How is the nasal dose administered? Are the animals constrained in a chamber? Is the nasal dose administered over a duration?

**Response:** For the oral dose of bromelain solution (40 mg/kg), 400 µl of bromelain solution was administered by oral gavage. The vehicle of solution was distilled water. No pH adjustment of bromelain solution was done during study.

30 µl of bromelain solution at a dose of 10 mg/Kg was administered in each nostril in 30 s with the help of micropipette with 0.1 mm internal diameter at delivery place. Nasal dose was administered using intransal grip, each rat was restrained twice and held with their neck parallel to the floor while administering nasal dose.

As per reviewer’s suggestion changes have been made in manuscript and represented as “Animals of group I received bromelain solution orally (400 µl, 40 mg/Kg body weight) by oral gavage while second group received nasally (30 µl, 10 mg/ Kg body weight) in each nostril in 30 s with the help of micropipette with 0.1 mm internal diameter at delivery place. Blood samples (300 µl) were collected from rat tail vein in heparinised coated tubes at pre-dose (0.0), 0.5, 1, 2, 4 and 6 h post dose of bromelain solution. Nasal dose was administered using intransal grip, each rat was restrained twice and held with their neck parallel to the floor while administering nasal dose.

**Comment 3:** Table 1, the increased in AUC looks like to be caused by a decrease in elimination? It may not be due to increase in bioavailability alone. The improved in efficacy may be due to higher drug concentration in the lung at 0.5 hour.

**Response:** The decrease in elimination rate constant increased the t_1/2_ and mean residence time which contributed to increase in AUC i.e., systemic bioavailability. Nasal administration improved the drug concentration in lungs which might have improved therapeutic efficacy of bromelain.
**Comment 4:** Figure 1 – it looks like the substantial difference in the lung concentration is only observed at 0.5 hour. In Figure 1, labelling with “A” and “B” and “a” and “b” is confusing.

**Response:** As per reviewer’s suggestion, labeling has been changed in Figure 1.
